# Application and evaluation of whole-process nursing in venous thromboembolism patients

**DOI:** 10.3389/fpubh.2025.1578074

**Published:** 2025-06-16

**Authors:** Yaqian Diao, Yu Huang, Xue Cui, Xinhua Ma, Hanxiao Zhao, Muxi Chen, Zhuzhu Zhao, Ruijiao Gao

**Affiliations:** Department of Vascular Surgery, Department of Third Hospital of Hebei Medical University, Shijiazhuang, Hebei Province, China

**Keywords:** venous thromboembolism, Total process management, nursing care, inferior vena cava filter placement, complications

## Abstract

**Background:**

Venous thromboembolism (VTE) is a serious threat to patient health. Currently, its management faces the problems of lack of patient awareness, poor medical and nursing management, and lack of out-of-hospital management. The traditional care model is difficult to meet the demand, and the whole process care management model (WPCM) has become an important direction of research to optimise the care of VTE patients.

**Objective:**

To evaluate the effectiveness of this model in patients with VTE and provide a basis for standardised management.

**Methods:**

A total of 1,392 patients admitted to our hospital from March 2023 to December 2024 with thrombosis due to filter placement were divided into an experimental group and a control group according to the time of admission. The experimental group adopted the WPCM, and the control group received routine care. Comparison of psychosomatic status, quality of life, standardised anticoagulation, filter removal, complications, hospital stay and patient satisfaction during hospitalisation in both groups.

**Results:**

There were no significant differences between the two groups in terms of baseline data such as gender, age, BMI and some indicators, *p* > 0.05. However, the experimental group had lower intrafilter thrombosis, incidence of PTS, length of hospital stay, average hospital costs and higher patient satisfaction than the control group, *p* < 0.05.

**Conclusion:**

The whole process management nursing mode is of great benefit to patients with venous thromboembolism. It can effectively reduce the incidence of in-filter thrombosis and PTS (post-thrombotic syndrome), shorten hospital stays, reduce costs and improve patient satisfaction, making it worthy of widespread clinical application.

## 1 Introduction

Venous thromboembolism (VTE) is a serious and potentially fatal condition that consists mainly of deep vein thrombosis (DVT) and pulmonary thromboembolism (PTE). These diseases are a serious threat to human health and epidemiological studies show that 2.5 to 5% of the population will experience DVT at some point in their lives (1, 2). Once the disease develops, patients are at high risk of death or severe disability from pulmonary embolism or post-thrombotic syndrome if they are not treated promptly and correctly (3–5). Even with treatment, VTE has a high rate of recurrence in the first few months after initial onset, with a recurrence rate of about 7% at 6 months (6). In addition, more than 50% of patients develop post-thrombotic syndrome (PTS) within 2 years of DVT (7–11).

Currently, there are many challenges in the management of VTE patients that need to be addressed. In clinical practice, the management of VTE patients is mainly focused on the hospitalisation period, while pre-hospital prevention and post-hospital rehabilitation management are relatively weak, especially post-hospital follow-up, which appears to be inadequate. Furthermore, thrombotic patients in the hospital setting are often comorbid with multiple injuries and other underlying medical conditions and are spread across different clinical departments, making uniform and standardised management difficult to implement (10). This fragmented management model severely limits patients’ access to comprehensive and continuous care services, which in turn affects treatment outcomes and quality of recovery.

As healthcare concepts continue to evolve, the importance of managing patients throughout the entire process is becoming increasingly important. 2022 The National Nursing Career Development Plan (2021–2025) issued by the NHSRC clearly states that nursing should focus on the health needs of the general population, creating an all-round, full-cycle, high-quality nursing service system, NHSRC on the issuance of the National Nursing Career Development Plan (2021–2025) Notice. Under the guidance of this policy, innovative care models to ensure patient safety, the implementation of accountable holistic care and the development of continuity of care services have become popular research directions in nursing.

Our hospital has actively responded to this industry development trend, and since March 2024, we have been meticulously building a VTE patient the whole process care management model (WPCM), covering the three key stages of pre-hospital, in-hospital and post-hospital care. The aim of this innovative model is to provide personalised, comprehensive and safe care to VTE patients, thereby improving patient prognosis and the overall quality of care. In this paper, we will describe the specific application process of the WPCM and the clinical outcomes achieved, hoping that it can provide useful references and lessons for clinical nursing practice.

## 2 Materials and methods

### 2.1 Research object

The present study was a retrospective study of patients admitted to the hospital from March 2023 to December 2024 who had thrombosis with filter placement. According to the time of filter placement, 728 patients admitted for filter placement in thrombosis from March 2024 to December 2024 were used as the experimental group, and 664 patients admitted for filter placement in thrombosis from March 2023 to December 2023 were used as the control group. Inclusion criteria: (i) The patient’s symptoms are in accordance with the Guidelines for the Diagnosis and Management of DVT of the Lower Extremities (3rd edition); (ii) The patient has normal thinking and communication skills and is able to cooperate with the treatment; (iii) All patients and their families have been fully informed about the study, clearly know the purpose, methods, possible risks and benefits of the study, and voluntarily participate in this study based on their personal wishes. Exclusion criteria: (i) The patient has a serious mental illness or impaired consciousness; (ii) The patient is breastfeeding or pregnant; (iii) The patient is associated with a serious primary disease; (iv) The patient is associated with fresh wounds, active ulcers or acute lymphadenitis of the lower extremities, or other conditions that would interfere with this study.

### 2.2 Methods for managing the entire process of inferior vena cava filter placement in patients with DVT

#### 2.2.1 Study management

A study on the management of the whole process of placing inferior vena cava filter in patients with DVT: (i) pre-hospital management: community, outpatients lack of knowledge of inferior vena cava filter and thrombosis, especially some sedentary, bedridden, fracture pregnancy and other patients, the occurrence of thrombosis do not know, do not know how to prevent; (ii) in-hospital management: Personalised treatment and care of patients with thrombosis in hospitals is not detailed enough, especially in the clinic of thrombosis in other departments, thrombosis treatment and placement of inferior vena cava filters in the lack of complete process management of patients (iii) Post-hospital management: some patients with inferior vena cava filters could not take anticoagulant drugs as prescribed by the doctor after discharge from the hospital, and the filters could not be taken out within the specified time.

#### 2.2.2 The management team

The management team for DVT patients with inferior vena cava filter placement consists of the director of vascular surgery as the head of the thrombosis management team, the nurse manager as the deputy head, the vascular surgeon team and the nursing team as the medical and nursing team.

#### 2.2.3 Responsibilities of the team members

The responsibilities and division of labour of the team members are clear: the director and the head nurse are responsible for supervising the implementation of the WPCM of thrombosis patients; the medical team is responsible for the diagnosis and treatment of thrombosis patients; the nursing team is responsible for the nursing care of the patients; and the medical and nursing teams are jointly responsible for the follow-up management of thrombosis patients discharged from hospital, including the taking of medication, the removal of filters and the psychological situation of the patients.

#### 2.2.4 Team member training

Team members receive regular training in the following five areas (i) Standardised diagnosis and treatment for DVT patients with IVC filters, including medication and surgery, etc. (ii) Standardised nursing care for DVT patients with IVC filters, including ward management, patient positioning and ankle pump movement, etc. (iii) Standardised health education for patients with IVC filters (iv) Standardised follow-up for patients with IVC filters (v) Psychological care for patients with IVC filters Each patient was jointly assessed, treated, cared for and followed up by the medical and nursing team after admission to the group, and the patients’ information was collected. After the patients were enrolled in the group, the medical and nursing team jointly assessed, diagnosed, treated, cared for, followed up and organised the patients’ data.

### 2.3 Implementation of total process management for placement of inferior vena cava filters in patients with DVT

#### 2.3.1 Pre-hospital management

The medical and nursing staff of the department is divided into 4 groups, forming a popular science clinic group, each group consists of 3 doctors and 4 nurses, regularly visiting outpatient clinics, communities, nursing homes, etc., To carry out popular science and clinic activities, targeting patients with prolonged sedentary, bed-ridden, bone fracture, pregnant patients to carry out popular knowledge of thrombosis, produced a small video of popular knowledge, popular knowledge of thrombosis in the form of a performance, and regular grassroots lectures on thrombosis to make patients with thrombosis pay attention to thrombosis, recognise the danger of thrombosis, and know that patients can be prevented and treated. Patients with thrombosis attach importance to thrombosis, recognise the danger of thrombosis and know that patients can be prevented and treated.

#### 2.3.2 In-hospital management

(i) VTE and bleeding risk assessment for all DVT patients, more caution for those with high bleeding risk, standardised use of anticoagulants, increased anticoagulant education, use of PDCA management tools and *ad hoc* bleeding risk improvement. (ii) DVT patients in the ward should be managed by responsible doctors and nurses at the bedside, and a detailed management plan should be made for each patient, and small check-ups of medical and nursing integration should be carried out daily and large check-ups should be carried out weekly under the leadership of the director and head nurse, so that problems can be detected and corrected in time. (iii) For thrombosis patients from other departments in the ward, the medical team will conduct consultation and formulate detailed treatment plan, and the nursing team will follow up the patients according to the follow-up system, make records and formulate detailed nursing plan. (iv) Strengthen the construction of humanistic wards, grow small green plants for patients in the wards to give patients confidence in overcoming diseases, and prepare warm items such as popular science books, straws and small snacks to make patients feel warm. (v)Intervention room staff shift adjustment, the implementation of 365 days 24 h duty system, the opening of the “36,524 warm heart plan,” to enter the intervention room patients: handshake appeasement, verbal communication; warm quilt, wear warm footwear to do everything to meet the needs of patients in various aspects.

#### 2.3.3 Out-of-hospital management

For patients discharged from hospital after filter placement, the nursing team will conduct out-of-hospital telephone follow-up, supervise regular follow-up, ask whether patients are taking anticoagulation regularly, and ensure the safety of patients’ medication; for patients who have not removed the filters in time, the report will be made to the medical team to supervise the timely removal of the filters.

### 2.4 Research methods

A total of 1,392 patients were enrolled in this study, of whom 728 received full process management and 664 received usual care. Data on patients’ mental health during hospitalisation, the occurrence of complications during hospitalisation and patient satisfaction were collected to clarify the benefits of placing inferior vena cava filters in DVT patients under the full process management care model.

### 2.5 Assessment of mental state

In this study, the mental status of the patients was an important indicator of the general condition of the patients. We used the Self-rating depression scale (SDS) to assess the occurrence of depression during hospitalisation (12). Zung self-rating anxiety scale (SAS) was used to assess the occurrence of anxiety in patients during hospitalisation (13), see Tables 1, 2 for details.

**Table 1:** Zung self-rating anxiety scale (SAS).

Assessment items	None	Sometimes	Often	Ongoing
1. I feel more nervous and anxious than usual.	1	2	3	4
2. I’m scared for no reason.	1	2	3	4
3. I’m easily upset or frightened.	1	2	3	4
4. I feel like my body is broken into pieces, torn apart.	1	2	3	4
5. I think everything is fine, and nothing unfortunate will happen.	4	3	2	1
6. My hands and feet are shaking and trembling.	1	2	3	4
7. I suffer from headaches, head and neck pains and back pains	1	2	3	4
8. I feel weak and tired.	1	2	3	4
9. I feel calm and it’s easy to sit quietly.	4	3	2	1
10. I feel my heart beating fast.	1	2	3	4
11. I’m suffering from bouts of vertigo.	1	2	3	4
12. I have fainting spells or feel like I’m going to faint.	1	2	3	4
13. It’s easy for me to breathe in and out.	4	3	2	1
14. I have numbness and tingling in my hands and feet	1	2	3	4
15. I’m suffering from stomach pains and indigestion.	1	2	3	4
16. I have to pee a lot.	1	2	3	4
17. My hands and feet are often dry and warm	4	3	2	1
18. My face feels hot and red.	1	2	3	4
19. I fall asleep easily and sleep well through the night.	1	2	3	4
20. I have nightmares.	1	2	3	4

The scores of the 20 questions were added together to obtain the total score, which was multiplied by 1.25 and rounded to the nearest whole number to obtain the standard score. According to the Chinese normative results, the cut-off value of the SAS standardised score is 50 points, where 50–59 points are considered mild anxiety, 60–69 points are considered moderate anxiety, and more than 69 points are considered severe anxiety.

**Table 2:** Depression self-rating scale (SDS).

Assessment items	None	Sometimes	Often	Ongoing
1. I feel sullen and depressed.	1	2	3	4
2. I think morning is the best part of the day.	4	3	2	1
3. I’m going to cry or feel like crying.	1	2	3	4
4. I do not sleep well at night.	1	2	3	4
5. I ate as much as I usually do.	4	3	2	1
6. I’m as happy as I’ve ever been in close contact with the opposite sex.	4	3	2	1
7. I found that I was losing weight.	1	2	3	4
8. I suffer from constipation.	1	2	3	4
9. Heart beating faster than usual.	1	2	3	4
10. I was tired for no reason.	1	2	3	4
11. My mind is as clear as usual.	4	3	2	1
12. I do not find it difficult to do things on a regular basis.	4	3	2	1
13. I feel restless and calm.	1	2	3	4
14. I have hope for the future.	4	3	2	1
15. I’m more angry and agitated than usual.	1	2	3	4
16. I think it’s easy to make a decision	4	3	2	1
17. I feel useful. Someone needs me.	4	3	2	1
18. I’ve had an interesting life.	4	3	2	1
19. I think if I die, someone else will have a better life.	1	2	3	4
20. Things that normally interest me still do.	`	3	2	1

The individual scores for each of the 20 items are added together to give a total score. The upper normal reference value for the total score is 41 points, and the standardised score is equal to the total score multiplied by 1.25 to the nearest whole number. The smaller the score, the better. The standardised upper limit of normal reference score is 53, 0–53: no symptoms of depression, 53–62: possible mild depression, 63–72: possible moderate depression, 72 or more: possible severe depression.

### 2.6 Patient access to treatment

Patients were treated in accordance with relevant guidelines and diagnostic and therapeutic standards during their hospital stay. Thirty-five of the enrolled patients did not receive standard anticoagulation therapy, including 2 patients with abdominal bleeding, 11 patients with contraindications to anticoagulation (platelets < 50 × 10^9^/L, road haemorrhage, etc.), 4 cerebral haemorrhage, 2 splenic haemorrhage, 4 gastrointestinal tract haemorrhage, and 12 cases of subarachnoid haemorrhage.

### 2.7 Complications in this study

Three types of complications were counted in this study: anticoagulant bleeding, intrafilter thrombosis and PTS. A total of 3 patients had anticoagulant haemorrhage manifested as haemoptysis. There were 21 cases of intrafilter thrombosis and 39 cases of PTS.

### 2.8 Statistical methods

The data were statistically analysed using SPSS 25.0 statistical software. Measurement data is represented by average number±standard deviation. Categorical variables are expressed as counts (percentages). When the measurement data obey the normal distribution, the paired t test is used for analysis. When the measurement data does not obey the normal distribution, it is represented by median (M) and interquartile interval (Q), and the rank sum test is used. Descriptive statistical analysis as well as one-way ANOVA were used to statistically analyse the indicators related to the adoption of total process management and routine care models. A chi-square test was conducted to examine the value of adopting a total process management and usual care model in reducing patient costs during hospitalisation and optimising the patient experience, *p* < 0.05 was considered a statistically significant difference.

## 3 Results

### 3.1 Comparison of baseline characteristics of patients in group

The two groups were balanced and comparable in terms of baseline characteristics such as sex, age and BMI, with a *p* value of less than 0.05. See Table 3 for details.

**Table 3:** Comparison of baseline characteristics.

Variables	Total (*n* = 1,392)	Control group (*n* = 664)	Experimental group (*n* = 728)	Statistic	*p*
Age	61.33 ± 15.48	61.33 ± 15.54	61.33 ± 15.45	*t* = −0.00	0.999
BMI	25.38 ± 3.93	25.39 ± 3.93	25.38 ± 3.93	*t* = 0.02	0.987
Gender, n (%)				χ^2^ = 0.02	0.896
Female	600 (43.10)	285 (42.92)	315 (43.27)		
Male	792 (56.90)	379 (57.08)	413 (56.73)		

### 3.2 Comparison of general characteristics of patients hospitalised in the two groups

After the patients were admitted to the hospital, the patients’ mental state and quality of life scores were counted and examined separately to exclude the influence of such factors on the final results. At the same time, all patients enrolled in the study received standardised diagnostic and treatment services according to clinical diagnostic and treatment norms to ensure consistency of the diagnostic and treatment process and to reduce the impact of individual differences on the results of the study. There was no significant difference between the two groups in terms of SAS, SDS scores, quality of life scores, standardised anticoagulation and standardised filter removal (*p* > 0.05), as shown in Table 4.

**Table 4:** Comparison of general characteristics of patients hospitalised.

Variables	Total (*n* = 1,392)	Control group (*n* = 664)	Experimental group (*n* = 728)	Statistic	*p*
Anxiety Self-Rating Scale (SAS)	50.04 ± 4.66	50.03 ± 4.75	50.04 ± 4.57	*t* = −0.06	0.951
Depression Self-Rating Scale (SDS)	51.33 ± 5.27	51.37 ± 5.26	51.30 ± 5.28	*t* = 0.24	0.810
Quality of life score	0.05 ± 0.22	0.05 ± 0.21	0.05 ± 0.22	*t* = −0.34	0.733
Regulate the removal of filters, n (%)				χ^2^ = 0.12	0.730
No	337 (24.21)	158 (23.80)	179 (24.59)		
Yes	1,055 (75.79)	506 (76.20)	549 (75.41)		
Standardised anticoagulation, n (%)				χ^2^ = 0.01	0.917
No	35 (2.51)	17 (2.56)	18 (2.47)		
Yes	1,357 (97.49)	647 (97.44)	710 (97.53)		

### 3.3 Comparison of complications between the two groups

The occurrence of complications in both groups was included in the statistical study and it was found that there was no statistical difference in the occurrence of intrafilter thrombosis and PTS during hospitalisation between the two groups, with the experimental group being superior to the control group. Anticoagulated bleeding was not statistically significantly different due to the small sample size of bleeding patients in both groups (*p* > 0.05). See Table 5 for details.

**Table 5:** Comparison of complications.

Variables	Total (*n* = 1,392)	Control group (*n* = 664)	Experimental group (*n* = 728)	Statistic	*p*
Bleeding after anticoagulation, n(%)				χ^2^ = 0.00	1.000
No	1,389 (99.78)	663 (99.85)	726 (99.73)		
Yes	3 (0.22)	1 (0.15)	2 (0.27)		
Intrafilter thrombosis, n(%)				χ^2^ = 4.81	**0.028**
No	1,371 (98.49)	649 (97.74)	722 (99.18)		
Yes	21 (1.51)	15 (2.26)	6 (0.82)		
PTS, n(%)				χ^2^ = 7.46	**0.006**
No	1,353 (97.20)	637 (95.93)	716 (98.35)		
Yes	39 (2.80)	27 (4.07)	12 (1.65)		

Bold values indicate a *P*-value less than 0.05, indicating statistical significance.

### 3.4 Comparison of hospitalisation in the two groups

Comparison showed that the average length of stay and average cost of hospitalisation of patients in the experimental group decreased significantly compared with those in the control group, and the difference was statistically significant (*p* < 0.05). See Table 6 for details.

**Table 6:** Comparison of hospitalisation.

Variables	Total (*n* = 1,392)	Control group (*n* = 664)	Experimental group (*n* = 728)	Statistic	*p*
Length of hospitalisation	19.06 ± 11.53	18.19 ± 11.68	19.84 ± 11.33	*t* = −2.68	**0.007**
Hospitalisation costs	67181.86 ± 51599.20	63511.88 ± 52276.13	70529.21 ± 50778.86	*t* = −2.54	**0.011**

Bold values indicate a *P*-value less than 0.05, indicating statistical significance.

### 3.5 Comparison of patient evaluations between the two groups

Patient satisfaction was significantly higher in the experimental group (96.98%) than in the control group (94.88%), and the experimental group also received more brocade flags than the control group, with statistically significant differences (*p* < 0.05). The increase in satisfaction is closely related to the patients’ positive assessment of treatment outcomes and hospital experience. See Table 7 for details.

**Table 7:** Comparison of patient evaluations.

Variables	Total (*n* = 1,392)	Control group (*n* = 664)	Experimental group (*n* = 728)	Statistic	*p*
Patient satisfaction, n(%)				χ^2^ = 3.96	**0.047**
No	56 (4.02)	34 (5.12)	22 (3.02)		
Yes	1,336 (95.98)	630 (94.88)	706 (96.98)		
Number of banners, n(%)				χ^2^ = 4.30	**0.038**
No	1,296 (93.10)	628 (94.58)	668 (91.76)		
Yes	96 (6.90)	36 (5.42)	60 (8.24)		
Number of complaints, n(%)				χ^2^ = 1.06	0.303
No	1,366 (98.13)	649 (97.74)	717 (98.49)		
Yes	26 (1.87)	15 (2.26)	11 (1.51)		

Bold values indicate a *P*-value less than 0.05, indicating statistical significance.

## 4 Discussion

### 4.1 Analysis of the advantages and effects of the application of the WPCM in patients with VTE

The Total Process Care Management model demonstrates several significant benefits in the treatment and rehabilitation process of patients with VTE. In terms of reducing the rate of intrafilter thrombosis, patients’ normative anticoagulation rate improved significantly after implementation of the programme, and the rate of intrafilter thrombosis was reduced from 2.26 to 0.82%, a significant decrease. This is largely due to the regular visits and personalised instructions that ensure that patients are able to follow their anticoagulation regimen to the letter, thereby ensuring high levels of anticoagulation adherence. In clinical practice, many patients are discharged from hospital with difficulties in managing their anticoagulation therapy correctly due to a lack of professional guidance, which significantly increases the risk of thrombosis. By providing a standardised follow-up mechanism, continuous monitoring and guidance of the patient’s anticoagulation therapy, Total Process Care Management was able to identify and correct potential problems in a timely manner, effectively reducing the risk of thrombosis in the filter.

Lower-extremity DVT, a common type of VTE, can present with limb swelling, discomfort and difficulty walking. Progression of DVT can also lead to death or severe disability due to pulmonary embolism, PTS or amputation (7). Inferior vena cava filters (IVCFs) are devices designed to prevent PTE caused by thrombus dislodgement from the inferior vena cava system, and early placement of an IVCF can effectively prevent the occurrence of PTE (14–16). In terms of reducing the incidence of post-thrombotic syndrome (PTS), although the specific mechanisms were not further explored in this study, it is hypothesised that the WPCM may have reduced the incidence of PTS through the synergistic effects of early thrombosis prevention, standardisation of anticoagulation therapy and patient rehabilitation advice. Standardised anticoagulation therapy effectively inhibits the development of thrombosis, while in out-of-hospital care, guidance to patients on rehabilitation exercises helps to improve lower limb circulation and reduce the risk of PTS. Improving patient satisfaction is also an important outcome of the WPCM. Through personalised education, patients have a deeper understanding of VTE and the importance of standardised anticoagulation after discharge, while standardised follow-up visits ensure continuity of care and give patients a sense of professional care at home. Personalised pre-operative education and psychological counselling reduced patients’ anxiety, and the intra-operative “36,524 Warm Heart Plan” further improved patients’ experience of medical care and increased their trust in the medical team, significantly increasing patient satisfaction and the banner rate.

### 4.2 Comparative analysis of the application of the WPCM in patients with VTE and existing studies

In the process of in-depth investigation of the application and evaluation of the effect of the WPCM in patients with VTE, this research team extensively reviewed a large amount of relevant literature. After a comprehensive comparative analysis, it was found that the WPCM proposed in this study is significantly innovative and unique. On the key issue of patient adherence to anticoagulation therapy, the results of the present study are highly compatible with those of the New Zealand and Vietnam studies, both of which found a high prevalence of self-interruption of medication by patients (17, 18). The New Zealand study found that up to 45% of the group of patients receiving anticoagulant medication stopped taking it within 12 months of starting treatment. This self-interruption of medication not only seriously compromises the therapeutic effect of VTE, but also greatly increases the risk of recurrence and worsening of the condition, highlighting the importance of standardised out-of-hospital management for VTE patients. However, there were significant differences when comparing traditional care management models, particularly group-based care management interventions (19, 20). Group-based care management can meet the basic care needs of patients to a certain extent, but it has many drawbacks when dealing with specific diseases such as VTE, which is complex, has a long treatment cycle and requires continuous management at several levels. Group-based care management makes it difficult to achieve seamless integration of pre-hospital, in-hospital and out-of-hospital management, resulting in a disconnect in the management of patients at different stages of treatment. This management disconnect can prevent patients from receiving consistent and coherent care guidance and support as they transition from hospital to home or other care settings, affecting the continuity and effectiveness of treatment. The lack of a robust and effective monitoring mechanism for group-based care management interventions makes it difficult to ensure that all care interventions are implemented rigorously and accurately, resulting in a significant reduction in the quality and effectiveness of care.

The WPCM of this study focuses on the integrated management of patients from pre-hospital to out-of-hospital, with a high degree of emphasis on the interface and synergy between the various stages. Through the establishment of a perfect follow-up system, medical staff can communicate with patients on a regular basis, keep abreast of their recovery progress, medication status and possible problems, and provide targeted guidance and support; through the establishment of an effective supervision mechanism, the various links in the care process can be strictly controlled to ensure that all care measures can be effectively implemented, thus ensuring that patients receive comprehensive and personalised quality care services, maximising the promotion of patients’ recovery and improving treatment effects.

### 4.3 Limitations

This study has some limitations that cannot be ignored. The relatively small sample size is one of the main problems. A small sample size may not be able to cover all types of VTE patients, including different ages, genders, disease severity and comorbidities, which limits the generalisability and reliability of the study results. There may be differences in the response of different patient groups to the total process care management model and these differences may not have been adequately captured in this study. The relatively short duration of the study only allowed us to observe the impact of the total process care management model on patients in the short term, and could not fully assess its role in the long-term prognosis of patients. Patients with VTE may experience recurrences or other complications months or even years after treatment, and short-term studies cannot accurately capture this information, to the detriment of a full understanding of the long-term effects of the modality. In addition, this study was retrospective, lacked randomisation and had more potential confounding bias. Moreover, this study performed time period-based grouping, which could be a potential confounder due to differences between time periods.

### 4.4 Future research directions

Future research could be conducted in the following directions. In terms of increasing the sample size, data on VTE patients from different regions and medical institutions should be widely collected to ensure that the sample is sufficiently representative to reflect the characteristics of different patient groups. By conducting a multicentre study to include more complex cases, we will conduct an in-depth analysis of the effectiveness of applying the total process care management model in different situations and develop more targeted management strategies. To extend the observation period and establish a long-term follow-up mechanism to track the long-term recovery of patients after treatment, including the rate of thrombus recurrence, changes in quality of life and the long-term impact on patients’ mental health. To investigate the applicability of the WPCM at different stages of the disease, for example, acute, sub-acute and chronic phases; there are differences in the care needs of patients and we should explore how to tailor the care programme according to the stage of the disease to achieve precise care. To study the response of different patient groups to the total process care management model, such as older adult patients and patients with other underlying diseases, etc., to analyse their characteristics and needs, and to further optimise the management model to provide more efficient and comprehensive care services for VTE patients.

## 5 Conclusion

In conclusion, there is still a long way to go in the prevention and treatment of VTE as a disease with high morbidity and mortality. In this study, the WPCM was applied to VTE patients, and the WPCM can significantly reduce the complication rate, shorten the length of stay, reduce the average cost of hospitalisation and improve patient satisfaction in VTE patients. This provides new ideas and methods for the clinical care of VTE patients and is of great value for promotion. However, this study still has some limitations, such as a small sample size and a short follow-up period. Future studies can further increase the sample size, extend the follow-up period, and explore the application effect of the WPCM in different types of VTE patients, so as to provide a more scientific and effective basis for the prevention and treatment of VTE.

## Data Availability

The original contributions presented in the study are included in the article/supplementary material, further inquiries can be directed to the corresponding author.
